# Editorial: The role of water stress and soil texture on plant roots anatomy, architecture, and senescence

**DOI:** 10.3389/fpls.2024.1490001

**Published:** 2024-11-07

**Authors:** Annie Irshad, Husain Ahmad, Izhar Muhammad, Sana Ullah Khan, Sajjad Raza

**Affiliations:** ^1^ School of Biosciences, University of Nottingham, Sutton Bonington, United Kingdom; ^2^ College of Horticulture, Northwest A&F University, Yangling, Shaanxi, China; ^3^ State Key Laboratory of Crop Stress Biology in Arid Areas, College of Life Sciences, Northwest A&F University, Yangling, Shaanxi, China; ^4^ The Queensland Alliance for Agriculture and Food Innovation (QAAFI), The University of Queensland, St Lucia, Brisbane, QLD, Australia; ^5^ School of Geographical Sciences, Nanjing University of Information Science & Technology, Nanjing, Jiangsu, China

**Keywords:** water stress, drought, soil texture, root architecture, abiotic stress

Roots are the primary plant organs that play a crucial role in various biological functions, including the uptake and transport of water and nutrients from the soil to the aboveground plant structures. The root system operates by penetrating the soil with the primary root and extending lateral roots to access water and nutrients (Rogers and Benfey, 2015). A well-established root network provides structural support and anchorage to plants (Aroca et al., 2005; Hamanishi and Campbell, 2011). Plants are exposed to a range of biotic and abiotic stresses, and roots, being in direct contact with the soil are the first ones to encounter belowground stresses (Amtmann et al., 2022). Given the critical role of roots in stress tolerance, understanding how specific factors like water availability and soil texture influence root development is essential. This Research Topic of Frontiers in Plant Science, titled “The Role of Water Stress and Soil Texture on Plant Roots Anatomy, Architecture, and Senescence,” brings together cutting-edge research exploring how these factors impact root growth and function.

Water stress characterized mainly by shortage (drought) or excess of water (waterlogging) severely affect plants growth. Globally, droughts have caused an estimated loss of 1820 million Mg in cereal (wheat, rice, and maize) production during the past four decades (Lesk et al., 2016). The expected reduced freshwater availability by 50% mainly due to climate change and the doubling of agricultural water demand by 2050 will cause widespread water stress conditions limiting root growth and crop yields in the future (Gupta et al., 2020).

Roots undergo several adaptive changes, such as altering their architecture and anatomy, to cope with stressed environment. Plants in response to drought close their stomata to limit water loss and this results in reduced carbon uptake via photosynthesis, eventually leading to reduced crop yields.

Drought minimizes nutrient accessibility and restricts root growth, and thereby has been widely reported to reduce crop yields (Zhan et al., 2015). A meta-analysis reported that drought significantly declined root length density and root depth by 11% and 38%, respectively (Zhou et al., 2018). Cope et al. found reduction in root depth under drought stress by 36% and 44% in soils which were previously used for the growth of wheat and oilseed rape, respectively. Moreover, the root system in oilseed rape grown soils was narrower with reduced total root length and convex hull area compared to wheat soils (Cope et al.). These findings provide valuable insights into how changes in soil conditions, influenced by previous crop types, can affect root growth under drought stress. This underscores the potential of crop diversification to promote adaptive root traits, enhancing resilience to drought. Carvalho et al. compared the growth of 45 popcorn F1 hybrids under drought stress, which were developed from 10 S7 lines by diallel mating scheme. Water stress decreased the root network area by 17%, root network length by 18%, and root network volume 19%. Carvalho et al. further stated that breeding for adaptation to drought stress in popcorns can be successful in hybrids at the early stages because of dominant genetic effects which control the growth traits at the seedling stage. Besides drought, excess water conditions driven mainly by heavy rainfall can also cause flooding and waterlogging in poorly drained soils. According to Shabala (2011), globally ~10–12% of the agricultural land is affected by waterlogging causing substantial crop yield losses. Waterlogging conditions cause the death of seminal roots and reduces root mass, and increases the formation of adventitious root to receive oxygen in wheat and Arabidopsis (Thomson et al., 1990; Eysholdt-Derzsó and Sauter, 2019).

Soil texture – the relative proportion of sand, silt, and clay particles plays a critical role in influencing plant water availability, water use efficiency, and how plants respond to water stress. A balanced mixture of sand, silt, clay which provides good aeration, water retention, and nutrient availability promote root growth (Ahmad and Li, 2021). On the other hand, heavy-textured soils with high clay content are prone to compaction, particularly when wet, and their dense structure can limit root growth, making these soils challenging for root development. Conversely, sandy soils may also hinder root penetration due to their lack of water retention and nutrient availability. Ahmad and Li (2021) reported that root diameter and length tend to decrease in clay loamy soils, while root area, volume, diameter, and number are higher in loamy soils. Soils with impaired structure, often resulting in higher bulk density, can disrupt the root-shoot balance by requiring more energy for root growth, ultimately restricting root development (Strock and Lynch, 2020). Reduced root growth in compacted soils can exacerbate drought stress effects as access to water and nutrients becomes limited. Under such conditions, roots may adapt by altering their anatomy and architecture to mitigate drought stress (Brunner et al., 2015), although this comes at the cost of using more energy to search for scarce water and nutrients (Rouphael et al., 2012).

Significant attention is being paid in developing crops with root system architectures resilient to stressed environments by using targeted breeding and advanced genetic approaches. Farooqi et al. used genome-wide association studies technique and identified 1199 significant marker-trait associations (MTAs) for 17 root traits and two shoot traits in barley through genotyping-by-sequencing (GBS). Farooqi et al. found that the QTLs for root length and diameter overlapped with root volume and surface area, emerging as most suitable traits for selection and breeding of deeper-rooting barley varieties suitable for environmental adaptation, such as droughts. Jin et al. genotyped the root system architecture related traits in wheat by using the wheat 90K single-nucleotide polymorphism (SNP) array. Jin et al. identified six QTLs associated with wheat root architecture and developed two competitive allele-specific PCR markers that can be utilized in wheat breeding programs to achieve better root system and stable yields.

The present Research Topic provides updates on the interplay between water stress, soil texture, and root development, which is essential for advancing our understanding of plant resilience in increasingly variable environments. Future studies should focus on integrating advanced imaging techniques and non-invasive technologies to explore root architecture and anatomy *in situ*, overcoming the challenges posed by soil opacity and heterogeneity. Additionally, expanding the use of genome-wide association studies technique and other molecular tools will be crucial for identifying key genetic traits associated with root adaptation to water conditions and serve as a foundational step towards gene cloning and enhancement of root architecture of crops. Collaborative efforts between agronomists and geneticists will be critical in translating these insights into practical strategies that ensure sustainable agricultural productivity in the face of climate change.
